# Short-Term Clinical Outcomes of Mesotherapy vs. Saline Injection in Patients with Lateral Epicondylitis: A Retrospective Comparative Study

**DOI:** 10.3390/jcm15218339

**Published:** 2026-10-26

**Authors:** Arzu Dinç Yavaş, Sümeyye Akçay

**Affiliations:** 1 Department of Physical Medicine and Rehabilitation, Faculty of Medicine, İstanbul Aydın University, Küçükçekmece, 34295 İstanbul, Türkiye; 2 Department of Occupational Therapy, Faculty of Health Sciences, Fenerbahçe University, Atatürk Mah., Ataşehir Bulvarı, Metropol İstanbul, Ataşehir, 34758 İstanbul, Türkiye

**Keywords:** tennis elbow, hand strength, pain measurement, trigger points, injections, intradermal

## Abstract

**Background:** Lateral epicondylitis (tennis elbow) is a common cause of lateral elbow pain and upper-extremity dysfunction. Mesotherapy, a minimally invasive local injection technique, has shown promising results in musculoskeletal pain, but comparative data versus saline injection in lateral epicondylitis remain scarce. This study compared clinical outcomes of mesotherapy and saline injection in patients with lateral epicondylitis. **Methods:** In this retrospective, non-randomized comparative study, medical records of 55 patients with lateral epicondylitis who received mesotherapy (*n* = 28) or saline injection (*n* = 27) in routine clinical practice were reviewed. Pain intensity (Visual Analog Scale, VAS), grip strength, pinch strength, and upper-extremity function (QuickDASH) were assessed at baseline and 15 days after the first treatment session, with comparisons adjusted for baseline values, age, sex, and symptom duration. **Results:** Both groups showed significant within-group improvement in VAS, pinch strength, and QuickDASH scores, while grip strength improved significantly only with mesotherapy. VAS reduction was significantly greater with mesotherapy (mean change 4.25 ± 2.08 vs. 2.48 ± 1.67; *p* = 0.001; Hedges’ g = 0.92), as was grip strength improvement (median change 4.00 vs. 1.00; *p* < 0.001; r = 0.48); both remained significant after adjustment (*p* < 0.001 and *p* = 0.001). No significant between-group differences emerged for pinch strength (*p* = 0.461) or QuickDASH (*p* = 0.893). **Conclusions:** Mesotherapy was associated with significantly greater short-term improvement in pain intensity and grip strength than saline injection in lateral epicondylitis, without a clear advantage in pinch strength or patient-reported function. Given the non-randomized design and short follow-up, these findings should be considered preliminary and require confirmation in prospective, randomized, saline-controlled trials with longer follow-up.

## 1. Introduction

Lateral epicondylitis, also known as tennis elbow, is a musculoskeletal condition characterized by lateral elbow pain and upper-extremity dysfunction [1,2]. Its prevalence in the general population has been estimated at approximately 1–3% [3]. It occurs most frequently in middle-aged adults [3]. Typical clinical features include tenderness over the lateral epicondyle, pain provoked by gripping or resisted wrist extension, reduced grip strength, and limitations in daily activities [1,2]. Although not life-threatening, lateral epicondylitis can cause substantial upper-extremity disability and interfere with work performance and daily activities [3,4].

Historically considered an inflammatory tendinitis, lateral epicondylitis is now more accurately described as a degenerative tendinopathy. Histopathological studies have demonstrated disorganized collagen structure, fibroblastic proliferation, vascular hyperplasia, and angiofibroblastic changes, with limited evidence of classic acute inflammatory cell infiltration [5,6]. The extensor carpi radialis brevis tendon is most commonly involved, and repetitive mechanical loading or overuse is thought to contribute to microtrauma, failed tendon healing, and degenerative changes at the common extensor tendon origin [2,7]. These pathophysiological features may explain not only pain but also reduced grip performance and functional limitations in affected patients.

Management options for lateral epicondylitis include patient education, activity and load modification, exercise-based physiotherapy, physical modalities, manual therapy, orthoses, non-steroidal anti-inflammatory drugs, and various injection-based interventions [1,2,8]. Although many patients improve with conservative care, symptoms may persist in a subset of individuals. In such cases, injection-based interventions may be considered as nonsurgical treatment options, whereas surgery is generally reserved for persistent symptoms that do not respond to appropriate conservative management [2,7]. However, despite the availability of multiple treatment approaches, the optimal management strategy remains controversial because of heterogeneous evidence and the lack of consistent superiority of any single intervention.

A range of injection- and needle-based interventions, including corticosteroid injection, platelet-rich plasma, prolotherapy, ozone injection, and dry needling, have been evaluated in patients with lateral epicondylitis [9,10,11,12,13]. However, in injection studies, it may be difficult to distinguish the specific pharmacological effect of the injected agent from the effects of needle stimulation, local mechanical stimulation, and contextual or placebo-related responses. Saline or placebo injections have also been associated with improvements in pain and function in patients with lateral epicondylitis, and in some studies these changes have reached clinically meaningful levels [14,15,16,17]. These findings indicate that saline injection should not be regarded as an inert placebo; rather, the injection procedure itself may exert mechanical, needling-related, contextual, or other nonspecific therapeutic effects that may contribute to clinical improvement [14,15,16,17]. Accordingly, saline injection was evaluated in the present study not as a placebo control but as a distinct local treatment used in clinical practice, allowing a comparison of clinical outcomes between two active local interventions for lateral epicondylitis.

Mesotherapy is a minimally invasive local treatment technique involving the administration of small doses of pharmacological agents through superficial intradermal or subcutaneous injections near the painful region [18,19]. In musculoskeletal pain conditions, mesotherapy is intended to provide local analgesic and anti-inflammatory effects, modulate local pharmacological responses, and reduce systemic drug exposure by delivering low-dose agents directly to the symptomatic area [18].

Mesotherapy has been evaluated in several musculoskeletal pain conditions; however, the overall evidence remains limited and methodologically heterogeneous, largely because most available studies are small, uncontrolled, and lack an inert or saline comparator [18,20]. Evidence specifically concerning lateral epicondylitis is even more limited: existing reports are uncontrolled observational studies that describe pain and patient-reported functional outcomes after mesotherapy alone, without a comparison group, and therefore cannot distinguish a specific treatment effect from natural recovery, needling-related stimulation, or contextual response [21,22]. Furthermore, clinical assessment in lateral elbow tendinopathy should not rely solely on pain scores; objective performance measures, particularly grip strength, are also important for evaluating physical function [23]. Patient-reported measures such as the QuickDASH may complement pain and strength assessments by capturing upper-extremity symptoms and limitations in daily activities [24]. To our knowledge, no previous study has directly compared mesotherapy with saline injection in patients with lateral epicondylitis while simultaneously evaluating pain intensity, objective grip and pinch strength, and patient-reported upper-extremity function.

Accordingly, this study aimed to compare the clinical outcomes of mesotherapy and saline injection in patients with lateral epicondylitis using pain intensity, grip strength, pinch strength, and upper-extremity function as outcome measures. We hypothesized that patients who had received mesotherapy would demonstrate greater clinical improvement than those who had received saline injection in pain intensity, grip strength, pinch strength, and upper-extremity function.

## 2. Materials and Methods

### 2.1. Study Design

This study was designed as a retrospective comparative study. The study protocol was approved by the Non-Interventional Clinical Research Ethics Committee of Istanbul Medipol University (Institutional Review Board approval no: 1275; date of approval: 30 October 2025). All procedures were conducted in accordance with the principles of the Declaration of Helsinki.

Medical records of patients treated between March and September 2025 were retrospectively reviewed for eligibility. All consecutive patients treated during this period were screened, and those meeting the predefined inclusion criteria and none of the exclusion criteria were included in the study. Patients were excluded if their medical records were incomplete or if the data required for the study outcomes were unavailable. The study period was selected based on the availability of medical records and the predefined study timeframe. Patients with complete baseline and post-treatment clinical assessment data were included in the analysis. Since the study was based on anonymized retrospective clinical data and no additional interventions were performed for research purposes, the ethics committee waived the requirement for study-specific informed consent.

### 2.2. Sample Size and Patient Selection

Because this was a retrospective comparative study, no a priori sample-size or power calculation was performed. Instead, all eligible patient records meeting the predefined inclusion and exclusion criteria during the study period were reviewed and included in the analysis. Therefore, the sample size was determined by the availability of eligible records rather than by a prospectively calculated target sample size. During the study period, medical records of 74 patients with a diagnosis of lateral epicondylitis were initially screened for eligibility. Of these, 13 patients were excluded due to symptom duration of less than three months, and 6 were excluded due to incomplete baseline or follow-up clinical data. Consequently, a total of 55 patients with complete baseline and post-treatment data (28 in the mesotherapy group and 27 in the saline group) were included in the final analysis.

Before the procedures, patients underwent clinical assessment based on their symptoms, physical examination findings, and available medical records. Routine ultrasound imaging was not performed in all patients.

The clinical diagnosis of lateral epicondylitis (LE) primarily relies on a targeted medical history and standardized physical examination findings of painful local palpation, positive Cozen’s test, and positive Maudsley’s test. A systematic review explicitly analyzes the global diagnostic standards used in clinical trials, documenting that painful local palpation (46.8%), Cozen’s test (42.1%), and Maudsley’s test (22.2%) are the most consistently implemented clinical parameters worldwide [25].

The inclusion and exclusion criteria are presented in Table 1.

### 2.3. Groups

Patients were classified into two groups based on the injection treatment documented in their medical records. The mesotherapy group comprised 28 patients who had received mesotherapy, whereas the saline group comprised 27 patients who had received saline injection. Because of the retrospective design, treatment allocation was not performed for research purposes; rather, the groups were defined according to the treatments administered during routine clinical practice. Treatment selection was not randomized but was based on clinical judgment, patient preference, and the presence of hypersensitivity to mesotherapy components.

In our clinical practice, both intradermal mesotherapy and trigger point injection with saline solution are used as independent local treatment options for lateral epicondylitis, and patients who had received either treatment during routine care were retrospectively identified for this study. Trigger point injection with saline solution is specifically offered to patients with known hypersensitivity or allergy to the components of the mesotherapy solution, as well as to patients who prefer not to receive a pharmacological injection. This approach is supported by reports indicating that saline injections can produce statistically significant and clinically meaningful improvements in pain and function in patients with lateral epicondylitis [17], and is further supported by evidence specific to mesotherapy: a retrospective comparative study of intradermal mesotherapy for chronic spinal pain found that normal saline solution produced clinical improvement comparable to a pharmacological drug cocktail, and concluded that, given the costs and risks associated with active drugs, normal saline may represent a favorable option in cost–benefit terms for localized pain [26]. The therapeutic effect of trigger point saline injection has been attributed primarily to mechanical disruption of the palpable taut band produced by needle penetration and redirection, with the injected fluid volume playing a secondary, distending role rather than a pharmacological one [27]. Accordingly, the treatment administered to each patient (mesotherapy or trigger point saline injection) reflected the treating physician’s clinical judgment during routine practice—considering drug hypersensitivity, patient preference, and the cost and risks associated with pharmacological agents—rather than a randomized or protocol-driven allocation.

### 2.4. Treatment Procedures

**Figure 1:**
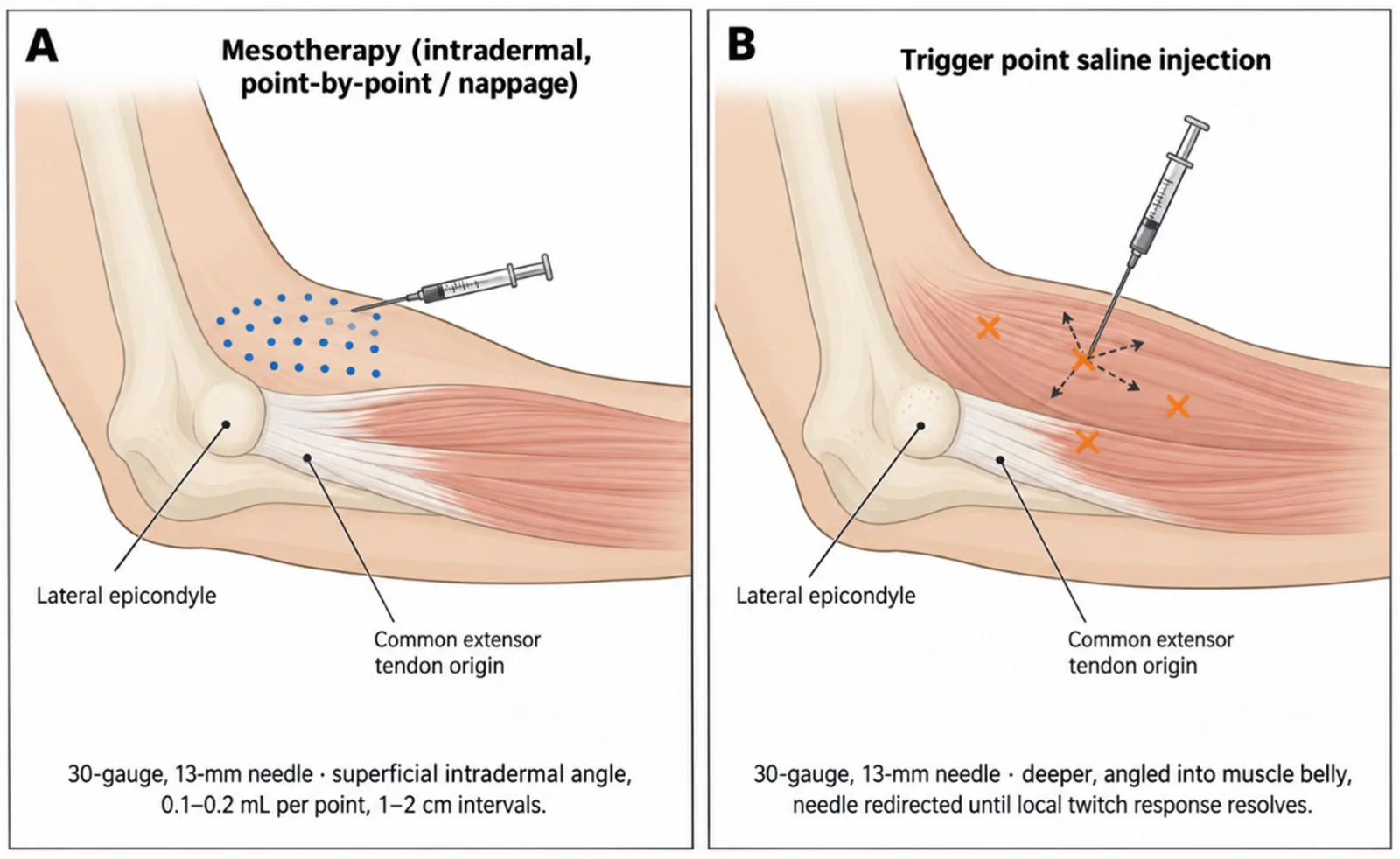
Schematic comparison of the two injection techniques. (**A**) Mesotherapy: intradermal, point-by-point (nappage) injections (0.1–0.2 mL per point, 1–2 cm intervals) over the lateral epicondyle and common extensor tendon origin. (**B**) Trigger point saline injection: deeper intramuscular injections at discrete, palpation-identified trigger points, with the needle withdrawn and redirected in multiple planes until the local twitch response resolved. Blue dots indicate individual mesotherapy injection points (**A**), and orange × marks indicate trigger point injection sites (**B**). Both techniques used a 30-gauge, 13-mm needle and a total injection volume of 4 mL per session. The illustration is a schematic representation for educational purposes and is not drawn to anatomical scale.

The injection procedure was performed under aseptic conditions by the same physician (A.D.Y) using a standardized technique. The injection site was determined according to the area of maximal tenderness over the lateral epicondyle and the common extensor tendon origin. In the mesotherapy group, a solution combining three agents commonly used in musculoskeletal pain management—lidocaine, thiocolchicoside, and meloxicam—was administered. Lidocaine 2% was first diluted 1:1 with 0.9% sodium chloride to yield a 1% solution, and 1 mL of this diluted solution was drawn into the syringe. Two milliliters of thiocolchicoside (4 mg/2 mL) were then added undiluted. Meloxicam (7.5 mg/1.5 mL) was separately diluted in 4 mL of 0.9% sodium chloride, and 1 mL of this dilution was incorporated into the mixture, giving a total injectable volume of 4 mL. This dilution protocol followed institutional practice and published mesotherapy preparation guidelines [28,29,30]. Injections were delivered intradermally using a point-by-point technique (nappage), depositing 0.1–0.2 mL per point at 1–2 cm intervals through a 30-gauge, 13-mm needle until discernible wheals appeared across the treatment area. In the saline group, trigger point injection with 0.9% sodium chloride was performed at palpable, painful trigger points identified by palpation over the affected forearm extensor musculature, rather than across a standardized skin area. A total volume of 4 mL of saline was administered per session using a 30-gauge, 13-mm needle, with the needle withdrawn and redirected in multiple planes at each trigger point until a local twitch response was no longer elicited (Figure 1) [27]. Although ultrasound guidance may improve anatomical localization and procedural precision, its additional clinical benefit for myofascial trigger point injections remains incompletely established. A systematic review by Diep et al. found that the available evidence supporting the superiority of ultrasound-guided over non-image-guided interventions was limited by high risk of bias and a lack of high-quality studies. Therefore, ultrasound guidance cannot be considered unequivocally necessary for every trigger point injection, although it may be particularly valuable when targeting deep muscles or structures close to important neurovascular anatomy [31].

This illustration was generated with the assistance of ChatGPT (GPT-5.6, OpenAI) and reviewed by the authors for accuracy.

The number of treatment sessions, injection volume, and treatment interval were the same in both groups according to the clinic’s treatment protocols. Both the mesotherapy and saline groups received four treatment sessions administered at 3-day intervals (days 1, 4, 7, and 10). For the mesotherapy group, this schedule was consistent with established mesotherapy protocols intended to achieve cumulative local drug effects while limiting systemic drug exposure [29]; the same session frequency was followed for the trigger point saline injections as part of the clinic’s standard treatment schedule for local injection therapy, irrespective of the specific agent administered. No additional intervention was performed for research purposes. In accordance with the clinic’s standard practice for lateral epicondylitis, all patients were advised on activity modification and provided a home-based exercise routine, along with recommendations to avoid wrist movements and heavy lifting likely to provoke symptoms. Formal physical therapy was not provided to either group during the treatment period, and analgesic use was restricted throughout the treatment course. Adverse events were retrospectively screened from the medical records, including post-injection pain exacerbation, swelling, erythema, infection, vasovagal reaction, allergic reaction, and any other procedure-related complication.

### 2.5. Outcome Measurements

The primary outcome measure was pain intensity, assessed using the Visual Analog Scale (VAS). Secondary outcome measures included grip strength, pinch strength, and upper-extremity function assessed using the QuickDASH questionnaire. All outcome measures were obtained from medical records at baseline and at post-treatment follow-up, 15 days after the first treatment session.

### 2.6. Demographic and Clinical Characteristics

Demographic and clinical characteristics were obtained from the medical records at baseline. These included age, sex, height, weight, body mass index (BMI), symptom duration, affected side, and dominant side.

### 2.7. Pain Assessment

Pain intensity was retrospectively extracted from the medical records. As part of routine clinical assessment, lateral elbow pain had been evaluated using a 10-cm Visual Analog Scale (VAS), anchored by “no pain” at 0 and “worst imaginable pain” at 10. Higher scores indicated greater pain intensity [32].

### 2.8. Grip and Pinch Strength Assessment

Grip strength and pinch strength values were retrospectively extracted from the medical records. Both measurements were obtained with participants seated, the shoulder adducted and neutrally rotated, the elbow flexed to 90°, the forearm in a neutral position, and the wrist supported at 0–30° of extension and 0–15° of ulnar deviation, consistent with standardized upper-extremity strength testing recommendations [33]. Grip strength was measured using a Jamar hydraulic hand dynamometer (Performance Health, Warrenville, IL, USA), and pinch strength was measured using a Baseline hydraulic pinch gauge (Fabrication Enterprises, Inc., White Plains, NY, USA), assessing tip pinch (thumb tip to index fingertip), the pinch pattern associated with maximal extensor muscle activation. For each trial, participants were instructed to exert maximal effort for approximately 3–5 s while standardized verbal encouragement was provided. Three consecutive trials were obtained with a 30–60-s rest interval between them to minimize fatigue, and the mean of the three trials was used for statistical analysis. All measurements were performed by the same examiner under consistent environmental conditions. Measurements were performed on the affected side, and higher values indicated better muscle strength and hand performance.

### 2.9. Upper-Extremity Function Assessment

Upper-extremity function and disability were retrospectively extracted from the medical records and assessed using the Quick Disabilities of the Arm, Shoulder and Hand questionnaire (QuickDASH). The QuickDASH is an abbreviated patient-reported outcome measure used to assess upper-extremity symptoms and activity limitations. Scores range from 0 to 100, with higher scores indicating greater symptom burden and functional limitation [24]. Therefore, a decrease in QuickDASH score from baseline to post-treatment was interpreted as functional improvement.

### 2.10. Statistical Analysis

Statistical analyses were performed using IBM SPSS Statistics for Windows, version 22.0 (IBM Corp., Armonk, NY, USA). The distribution of continuous variables was assessed using the Shapiro–Wilk test, together with visual inspection of descriptive distributional characteristics. Continuous variables were presented as mean ± standard deviation or median (interquartile range), depending on their distribution. Categorical variables were presented as frequencies and percentages. For the main treatment-effect table, both mean ± standard deviation and median (interquartile range) values were provided to improve transparency. All statistical tests were two-tailed, and a *p*-value < 0.05 was considered statistically significant.

Baseline demographic and clinical characteristics were compared between the mesotherapy and saline groups. An independent-samples *t*-test was used for normally distributed continuous variables, whereas the Mann–Whitney U test was used for non-normally distributed variables. Categorical variables were compared using the chi-square test or Fisher’s exact test, as appropriate. Effect sizes were reported as Hedges’ g for normally distributed continuous variables, r for non-parametric comparisons, and Cramer’s V for categorical variables.

Change scores were calculated for all clinical outcomes. For VAS and QuickDASH scores, change was calculated as baseline minus post-treatment values, whereas for grip and pinch strength, change was calculated as post-treatment minus baseline values. Thus, positive change values indicated clinical improvement for all outcomes. Within-group pre–post changes were analyzed using a paired-samples *t*-test for normally distributed change scores and a Wilcoxon signed-rank test for non-normally distributed change scores. Between-group comparisons of change scores were performed using an independent-samples *t*-test or Mann–Whitney U test, according to the distribution of the change scores. VAS change was defined as the primary outcome. For secondary outcomes, *p*-values were interpreted with consideration of the Holm–Bonferroni approach for multiple comparisons.

To evaluate adjusted between-group differences, univariate general linear models were constructed for each post-treatment outcome. In these models, the post-treatment outcome value was entered as the dependent variable, treatment group as the main independent variable, and the corresponding baseline outcome value, age, sex, and symptom duration as covariates. Adjusted mean differences with 95% confidence intervals, F statistics, *p*-values, and partial eta squared values were reported. Model assumptions, including homogeneity of error variances, were evaluated before interpretation. When the homogeneity of error variances assumption was violated, the corresponding model was additionally verified using heteroscedasticity-consistent (HC3) robust standard errors and a nonparametric bootstrap procedure with 5000 resamples, and the bootstrap-based 95% confidence interval and *p*-value were reported alongside the original model estimate.

Responder analyses were performed to assess clinically meaningful improvement. Clinically meaningful VAS improvement was defined as a reduction of at least 1.5 points [34,35], and clinically meaningful improvement in QuickDASH was defined as a reduction of at least 15.91 points, based on a previously reported minimal clinically important difference (MCID) estimate for patients with upper-extremity musculoskeletal disorders [36] from baseline to post-treatment. Responder rates were compared between groups using chi-square or Fisher’s exact test, as appropriate. In addition, Spearman correlation analysis was used to examine associations between symptom duration and clinical changes, as well as between changes in pain intensity and changes in grip strength, pinch strength, and QuickDASH scores. This correlation analysis was considered exploratory; because change-score correlations may be influenced by measurement error and regression to the mean and no adjustment for multiple comparisons was applied, the findings should be interpreted as hypothesis-generating rather than confirmatory.

## 3. Results

Baseline demographic and clinical characteristics of the participants are presented in Table 2. A total of 55 patients were included in the analysis, with 28 patients in the mesotherapy group and 27 patients in the saline group. The two groups did not differ significantly in BMI, affected side, dominant side, baseline pain intensity, grip strength, pinch strength, or QuickDASH scores. Although none of the baseline comparisons reached conventional statistical significance, age (*p* = 0.068), symptom duration (*p* = 0.064), and sex distribution (*p* = 0.050) showed borderline between-group differences, with patients in the saline group tending to be older, having a longer symptom duration, and including a higher proportion of female patients. Therefore, sex, age, symptom duration, and baseline outcome values were considered as potential covariates in the adjusted analyses. Given the relatively small sample size and non-randomized retrospective design, these baseline imbalances may be clinically relevant despite the absence of statistically significant differences.

**Figure 2:**
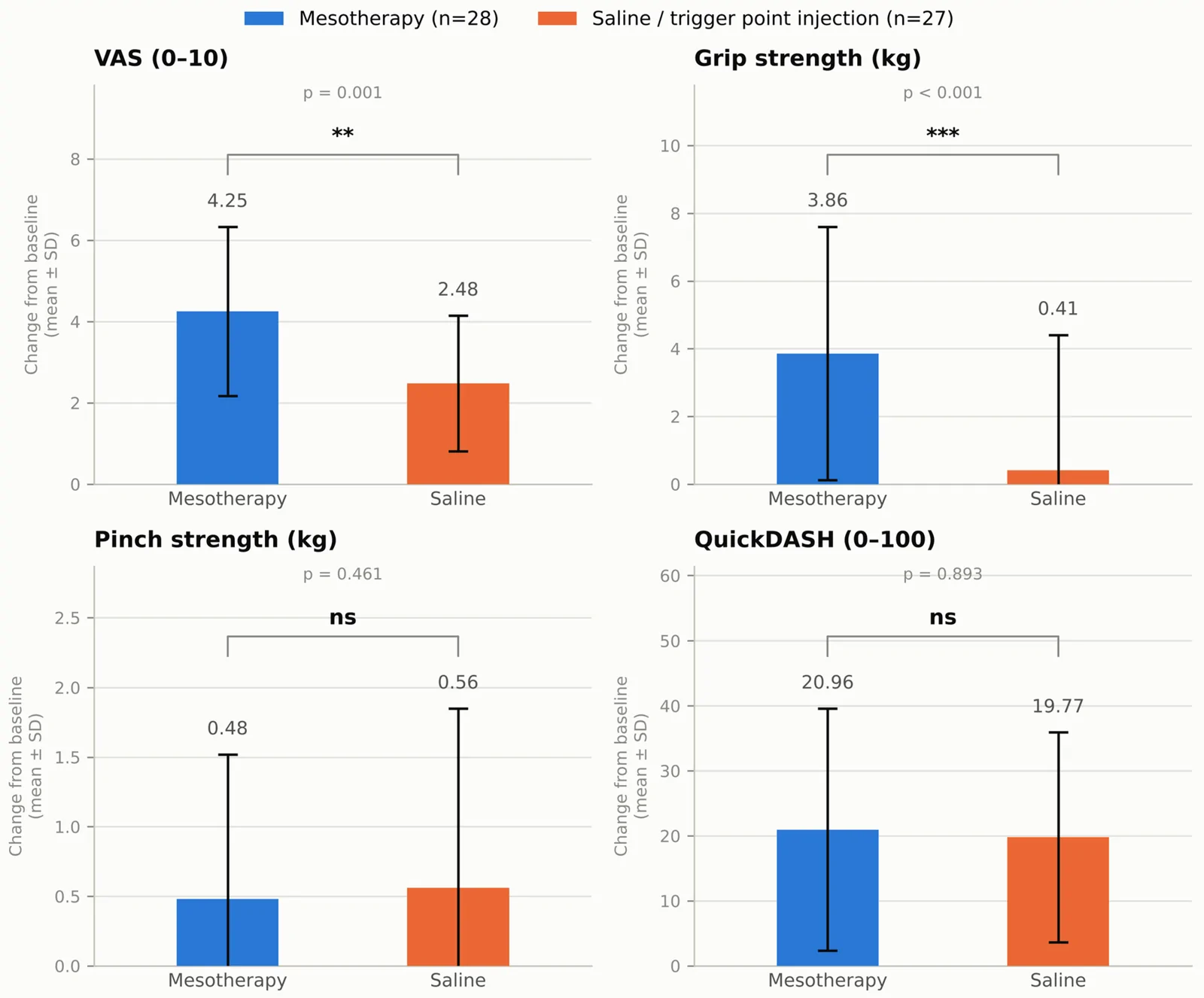
Change in clinical outcomes (mean ± SD change from baseline) by treatment group. VAS = Visual Analog Scale; QuickDASH = Quick Disabilities of the Arm, Shoulder and Hand. Error bars represent ±1 standard deviation. Statistical comparisons: independent-samples *t*-test for VAS change; Mann–Whitney U test for grip strength, pinch strength, and QuickDASH change. ** *p* = 0.001; *** *p* < 0.001; ns = not significant (*p* > 0.05). Mesotherapy, *n* = 28; saline/trigger point injection, *n* = 27.

Changes in clinical outcomes from baseline to post-treatment are summarized in Table 3 and illustrated in Figure 2. Both groups showed significant within-group reductions in VAS pain scores; however, the decrease was significantly greater in the mesotherapy group than in the saline group (mean change: 4.25 ± 2.08 vs. 2.48 ± 1.67; mean difference: 1.77, 95% CI: 0.74 to 2.79; t = 3.463, *p* = 0.001; Hedges’ g = 0.92).

Grip strength improved significantly within the mesotherapy group but not within the saline group, and the between-group comparison demonstrated a significantly greater improvement in the mesotherapy group (median change: 4.00 [2.00–6.00] vs. 1.00 [0.00–3.00]; U = 167.50, Z = −3.587, *p* < 0.001; r = 0.48). Although pinch strength and QuickDASH scores improved significantly within both groups, the magnitude of change did not differ significantly between groups for either pinch strength (*p* = 0.461) or QuickDASH (*p* = 0.893). The between-group difference in grip strength change remained significant after Holm–Bonferroni correction for secondary outcomes, whereas the between-group differences in pinch strength and QuickDASH change remained non-significant.

Adjusted between-group comparisons of post-treatment outcomes are presented in Table 4. After controlling for the corresponding baseline outcome value, age, sex, and symptom duration, treatment group remained significantly associated with post-treatment VAS score. The adjusted post-treatment VAS score was significantly lower in the mesotherapy group than in the saline group (adjusted mean difference: −1.91, 95% CI: −2.83 to −0.98; F = 17.189, *p* < 0.001; partial η^2^ = 0.260).

Similarly, post-treatment grip strength was significantly higher in the mesotherapy group after adjustment for baseline grip strength and covariates (adjusted mean difference: 4.22, 95% CI: 1.92 to 6.52; F = 13.610, *p* = 0.001; partial η^2^ = 0.217). Because the homogeneity of error variances assumption was not met for this model (Levene’s F = 2.98, *p* = 0.090), the result was verified using heteroscedasticity-consistent (HC3) robust standard errors and a nonparametric bootstrap procedure (5000 resamples), both of which confirmed a statistically significant adjusted between-group difference (HC3: 95% CI 1.60 to 6.84, *p* = 0.002; bootstrap: 95% CI 1.60 to 6.47, *p* = 0.002), consistent with the non-parametric change-score analysis (Supplementary Table S1).

In contrast, treatment group was not significantly associated with post-treatment pinch strength or QuickDASH score after adjustment for baseline values and covariates. The adjusted mean difference was −0.06 for pinch strength (95% CI: −0.73 to 0.61; F = 0.035, *p* = 0.853; partial η^2^ = 0.001) and −2.95 for QuickDASH score (95% CI: −12.00 to 6.10; F = 0.430, *p* = 0.515; partial η^2^ = 0.009).

The responder analysis based on clinically meaningful improvement is presented in Supplementary Table S2. The proportion of patients achieving clinically meaningful VAS improvement was significantly higher in the mesotherapy group than in the saline group (96.4% vs. 70.4%; Fisher’s exact *p* = 0.012; Cramer’s V = 0.352). In contrast, the proportion of patients achieving clinically meaningful QuickDASH improvement was numerically higher in the saline group than in the mesotherapy group, although this difference did not reach statistical significance (50.0% vs. 63.0%; *p* = 0.333; Cramer’s V = 0.131); thus, mesotherapy did not demonstrate superiority over saline injection with respect to patient-reported functional improvement.

Spearman correlation analyses are presented in Supplementary Table S3. A significant positive correlation was observed between VAS change and grip strength change (rho = 0.396, *p* = 0.003), indicating that greater pain reduction was associated with greater improvement in grip strength; however, given the exploratory nature of this analysis, this finding should be interpreted cautiously and does not establish a causal relationship.

### Safety Outcomes

No adverse events were documented in the available medical records during the follow-up period in either the mesotherapy or saline injection group. No serious complications, treatment discontinuations, or adverse events requiring additional medical intervention were identified.

## 4. Discussion

At the beginning of the study, we hypothesized that patients who had received mesotherapy would demonstrate greater clinical improvement than those who had received saline injection in pain intensity, grip strength and the other prespecified functional outcome measures.

The principal finding of this study was that mesotherapy was associated with significantly greater improvement in pain intensity and grip strength than saline injection in patients with lateral epicondylitis. Although both treatment groups demonstrated post-treatment clinical improvement in VAS, pinch strength, and QuickDASH scores, grip strength improved significantly only in the mesotherapy group, suggesting a potential additional therapeutic effect beyond the clinically meaningful improvements that have previously been reported after normal saline injection, including procedure-related, contextual, and needling-associated effects. In contrast, improvements in pinch strength and patient-reported upper-extremity function, as assessed by the QuickDASH, did not differ significantly between groups. These findings indicate that the clinical benefit of mesotherapy may be more evident in pain modulation and grip performance than in broader self-reported functional recovery over the observed follow-up period. Because treatment allocation was not randomized and outcome assessment was limited to 15 days after the first treatment session, these findings should be interpreted as an association rather than definitive evidence of a superior treatment effect, and the broader clinical implications should be considered preliminary.

Although within-group improvements were observed for some outcomes, these findings should be interpreted cautiously, as statistically significant changes within one group and nonsignificant changes within the other do not establish a statistically significant difference between treatments. Therefore, the comparative effects of mesotherapy and saline injection should be interpreted primarily on the basis of the between-group analyses.

These findings are consistent with the emerging but still preliminary evidence supporting the clinical use of mesotherapy in lateral epicondylitis. Temel et al. reported significant improvements in pain intensity and QuickDASH scores following mesotherapy in a retrospective cohort of patients with lateral epicondylitis, while Sampaio and Cruz reported improvements in pain, disability, work-related limitations, and sleep disturbance in a small uncontrolled case series [21,22]. However, because these studies lacked a saline comparison group and were not designed to isolate the specific treatment effect of mesotherapy from natural recovery, needling-related effects, or contextual responses, the present saline-comparative analysis may provide additional clinically relevant information.

Several possible mechanisms may underlie the greater pain reduction and grip strength improvement observed with mesotherapy. Beyond local effects on the common extensor tendon, the overlying fascia contains small-diameter nociceptive afferents (e.g., C fibers) that intradermal and subcutaneous injections may modulate, potentially contributing to pain reduction [37]; mesotherapy’s low-dose, localized drug delivery may also prolong local drug availability while limiting systemic exposure [18,19,38]. However, because the saline group also improved, procedure-related and needling-associated stimulation likely contributed as well [38,39,40], consistent with the view that lateral epicondylitis reflects a degenerative rather than purely inflammatory process [5,6]. A plausible interpretation is that reduced nociceptive input allowed greater grip force generation through decreased pain-related inhibition. As these mechanisms were not directly assessed, this discussion should be considered hypothesis-generating rather than confirmatory.

The greater improvement in grip strength in the mesotherapy group was statistically robust and accompanied by a large effect size; however, because no validated minimal clinically important difference for grip strength has been established specifically for lateral epicondylitis, this improvement should be described as a statistically and objectively meaningful change rather than one of confirmed clinical importance. Grip strength nonetheless remains a reliable and responsive objective measure in lateral epicondylitis, supporting its use as a clinical outcome measure. Smidt et al. demonstrated good interobserver reproducibility for pain-free and maximal grip strength measurements in patients with lateral epicondylitis, supporting their use as clinical outcome measures [41]. Stratford and Levy reported that pain-free grip strength was sensitive to clinical change over time, while Abbott et al. demonstrated an immediate improvement in pain-free grip strength following a single application of mobilization with movement [42,43]. In the present study, the parallel improvement in pain intensity and grip strength may indicate that pain reduction allowed patients to generate greater force during gripping tasks by reducing pain-related inhibition and improving load tolerance. This interpretation is further supported by the positive correlation between changes in pain intensity and grip strength, suggesting that patients with greater pain reduction tended to demonstrate greater gains in grip performance. These findings highlight the importance of including objective strength measures, in addition to pain scores, when evaluating clinical response in lateral epicondylitis.

In contrast to grip strength, pinch strength did not differ significantly between the mesotherapy and saline groups, although both groups showed within-group improvement after treatment. This finding may indicate that the beneficial effect of mesotherapy was more evident in forceful gripping than in more selective fine hand strength tasks, as extensor muscle activation is more pronounced during forceful grip than during fine pinch movements. In lateral epicondylitis, symptoms are typically provoked by gripping and resisted wrist extension, which place direct mechanical demand on the common extensor tendon origin [44]. Pinch performance relies substantially on coordinated thumb and finger muscle activity and interdigit force control [45,46]. Therefore, it is possible that pinch strength was less directly influenced by changes in lateral elbow pain than grip strength. However, this explanation remains hypothetical because the differential effects of lateral epicondylitis on grip and pinch strength have not been directly established. Accordingly, the greater pain reduction observed in the mesotherapy group may have translated more clearly into improved grip performance than into pinch strength. In addition, the relatively small sample size may have limited the ability to detect subtle between-group differences in pinch strength.

Although QuickDASH scores improved significantly within both groups, the magnitude of improvement did not differ significantly between the mesotherapy and saline groups. This finding suggests that the greater reductions in pain intensity and the greater gains in grip strength observed in the mesotherapy group did not fully translate into superior patient-reported upper-extremity function. One possible explanation is that the QuickDASH is a broad patient-reported measure of upper-extremity activity limitation and symptom burden rather than a direct measure of local elbow pain or isolated strength performance [24,47]. Therefore, localized improvements in pain and grip performance may not necessarily produce a detectable between-group difference in a multidimensional disability score, particularly over a limited follow-up period. In addition, improvements in pain and function have also been observed in saline or placebo groups in lateral epicondylitis injection trials, while active injection therapies have not demonstrated consistent superiority across patient-reported functional outcomes [48,49,50,51,52]. Psychosocial factors and coping-related variables may also influence perceived disability in lateral epicondylitis, further supporting the idea that pain and grip strength improvements may not correspond directly to proportional changes in patient-reported function [53]. Thus, although mesotherapy appeared to provide greater benefit in pain reduction and grip performance, this advantage may not have been sufficient to generate a distinct QuickDASH response compared with saline injection. Consistent with this, the proportion of patients achieving clinically meaningful QuickDASH improvement was numerically higher in the saline group than in the mesotherapy group, although this difference was not statistically significant, further supporting the absence of a clear functional advantage of mesotherapy over saline injection.

The improvement observed in the saline injection group should be interpreted as evidence that the comparison intervention was not inert. A systematic review and meta-analysis of 15 randomized controlled trials reported that normal saline injections produced statistically significant and clinically meaningful improvements in pain and function in patients with lateral epicondylitis, with a pooled mean reduction of 3.61 cm on the visual analog scale, and cautioned that such saline responses may obscure the true efficacy of active injectable therapies [17]. This interpretation is also supported by clinical trials showing improvement in saline or placebo arms. Wolf et al. reported that saline, corticosteroid, and autologous blood injections all resulted in improvement over 6 months, without a clear advantage of the active injections over saline [52]. Similarly, Krogh et al. found that pain decreased in corticosteroid, platelet-rich plasma, and saline groups, with no significant between-group difference at the primary follow-up point [48]. These findings suggest that improvement after saline injection may reflect a combination of needling-related tissue stimulation, contextual or placebo-related responses, regression to the mean, and the natural clinical course of lateral epicondylitis, rather than a specific pharmacological effect of saline itself. The significantly greater reductions in pain intensity and grip strength observed in the mesotherapy group in the present study, despite this expected saline-related improvement, are consistent with the hypothesis that mesotherapy may provide additional benefit beyond nonspecific injection-related responses; however, this observational, non-randomized comparison cannot establish a causal treatment effect, and this observation should be confirmed in prospective randomized saline-controlled trials. Therefore, the improvement in the saline group in the present study does not diminish the clinical relevance of the findings; instead, it strengthens the importance of saline-controlled comparisons when evaluating whether mesotherapy provides additional benefit beyond procedure-related effects.

The present findings may have several clinical implications. First, they support the importance of evaluating treatment response in lateral epicondylitis using both symptom-based and performance-based outcomes, rather than relying solely on pain intensity. The greater improvement in grip strength observed in the mesotherapy group suggests that pain reduction may be accompanied by clinically relevant gains in force generation during gripping tasks. However, because between-group differences in pinch strength and QuickDASH were not significant, mesotherapy should not be interpreted as producing uniform superiority across all domains of hand function or upper-extremity disability. From a clinical perspective, mesotherapy may be considered a potential local injection-based option for selected patients, particularly when the therapeutic goal is pain reduction and improvement in grip performance. Nevertheless, injection therapies should be viewed as adjunctive strategies rather than replacements for comprehensive management approaches, including education, load modification, exercise-based rehabilitation, and functional restoration.

This study has several strengths. First, it compared mesotherapy with saline injection rather than relying solely on a pre–post design, allowing a more clinically relevant interpretation of treatment-related changes beyond non-specific injection effects. Second, the study evaluated multiple outcome domains, including pain intensity, objective strength measures, and patient-reported upper-extremity function. This multidimensional assessment is important in lateral epicondylitis because clinical improvement may not be fully captured by pain scores alone. Third, the inclusion of grip strength and pinch strength provided objective performance-based information, while the QuickDASH reflected the patient-perceived impact of symptoms on upper-extremity function. Finally, the use of adjusted analyses and clinically meaningful responder analyses strengthened the interpretation of the findings.

This study also has several limitations. First, the retrospective design precludes causal inference, and treatment allocation was based on clinical practice records rather than randomization; therefore, selection bias cannot be excluded. Second, although the observed differences in baseline characteristics did not reach conventional levels of statistical significance, age showed a moderate effect size, symptom duration differed numerically, and sex distribution was borderline different between groups. In a small, non-randomized retrospective study, statistical non-significance does not necessarily indicate clinical equivalence, and these imbalances may have influenced the observed treatment effects. Although adjusted analyses were performed to account for these covariates, residual confounding cannot be fully excluded. Third, the sample size was relatively small, which may have limited the statistical power to detect subtle between-group differences, particularly for pinch strength and QuickDASH outcomes; therefore, the absence of statistically significant between-group differences for these outcomes should not necessarily be interpreted as evidence of equivalence between the two treatments. Fourth, the follow-up assessment was limited to a single post-treatment time point 15 days after the first treatment session, based on the available clinical records; longer-term outcomes, recurrence rates, and durability of treatment effects beyond this short follow-up window could not be evaluated. Fifth, although all patients received standardized activity-modification advice and a home exercise routine, and analgesic use was restricted during the treatment period, individual adherence to these recommendations could not be verified from the retrospective records, and this potential source of variability should be acknowledged. Sixth, trigger point injections were performed based on palpation without ultrasound guidance; although this reflects routine clinical practice, ultrasound guidance may improve anatomical localization and procedural precision, particularly for deep or anatomically sensitive injection sites, and its absence should be acknowledged as a methodological limitation. Finally, because all injection procedures were performed by a single author (A.D.Y.), the possibility of investigator-related bias in treatment delivery cannot be entirely excluded, although outcome measurements were retrospectively extracted from standardized medical records by the other author (S.A.), independent of treatment administration. Larger prospective, randomized studies with an a priori sample-size calculation, longer follow-up, and standardized protocols are needed to confirm these findings and provide more robust estimates of treatment effects.

Other most used treatment strategies for lateral elbow tendinopathy are Low-level laser therapy, extracorporeal shock wave therapy (ESWT), and progressive resisted exercises. Low-level laser therapy may provide short-term symptom relief when appropriate treatment parameters are used, although the evidence remains heterogeneous [54,55]. Similarly, the clinical effectiveness of ESWT remains uncertain, with systematic reviews reporting inconsistent comparative benefits [56,57]. Progressive resisted exercises targeting the wrist extensors are supported by clinical practice guidelines and should remain an important component of rehabilitation [58]. Mesotherapy may be considered as a potential adjunct to a multimodal rehabilitation program for lateral elbow tendinopathy. However, evidence directly demonstrating additional benefits from combining mesotherapy with laser therapy, ESWT, or exercise is currently insufficient. Future controlled studies should investigate whether such multimodal approaches improve pain, grip strength, and functional outcomes beyond those achieved with individual interventions.

For injection treatments, future prospective, randomized, saline-controlled trials with larger sample sizes are needed to confirm these findings. Such trials should also directly compare ultrasound-guided and palpation-guided injection techniques to determine whether image guidance improves procedural accuracy and clinical outcomes. Such studies should use standardized mesotherapy protocols, clearly define injection dose, content, technique, number of sessions, and treatment intervals, and include longer follow-up periods to evaluate the durability of clinical effects. Future research should also assess recurrence, adverse events, patient satisfaction, return to work, and rehabilitation adherence. Combining pain outcomes with objective measures such as pain-free grip strength and patient-reported outcomes such as QuickDASH or the Patient-Rated Tennis Elbow Evaluation (PRTEE) may provide a more comprehensive understanding of treatment response in lateral epicondylitis.

## 5. Conclusions

In conclusion, in this retrospective, non-randomized comparison, mesotherapy was associated with greater short-term improvement in pain intensity and grip strength than saline injection in patients with lateral epicondylitis. However, between-group differences in pinch strength and QuickDASH were not significant. Given the non-randomized design, the possibility of residual confounding, the limited sample size, and the short 15-day follow-up, these findings should be interpreted as a preliminary association rather than definitive evidence of a treatment effect beyond saline- or procedure-related responses, and confirmed by well-designed prospective randomized controlled trials with longer follow-up.

## Figures and Tables

**Table 1:** Inclusion and exclusion criteria.

Inclusion criteria
Age 20–75 years Diagnosis of lateral epicondylitis based on clinical examination Presence of lateral elbow pain and tenderness over the lateral epicondyle Positive Cozen’s Test Positive Maudrey’s Test Symptom duration of more than 3 months Receipt of mesotherapy or saline injection treatment Receipt of a minimum of four treatment sessions (mesotherapy or saline injection, as applicable) Failure to respond to initial conservative treatment (e.g., rest, oral NSAIDs, or physical therapy) Availability of complete clinical assessment records before treatment and after treatment Availability of baseline and post-treatment VAS, grip strength, pinch strength, and QuickDASH data
Exclusion criteria
Diagnosis of radial tunnel syndrome Diagnosis of cervical radiculopathy Previous elbow surgery on the affected side History of elbow fracture, dislocation, or major trauma Previous injection therapy to the affected elbow within the last 3 months Concomitant cervical radiculopathy, peripheral neuropathy, or other neurological disorder affecting the upper extremity Known hypersensitivity to any components of the mesotherapy solution Systemic inflammatory or rheumatologic disease Severe uncontrolled systemic disease Coagulation disorder or bleeding tendency Use of anticoagulant therapy that contraindicated injection treatment Pregnancy Incomplete or missing clinical records

**Table 2:** Baseline demographic and clinical characteristics of the participants.

Variable	Mesotherapy *n* = 28	Saline *n* = 27	*p*-Value	Effect Size
Age, years	49.11 ± 8.59	53.89 ± 10.37	0.068 ^a^	0.50
Sex, *n* (%)			0.050 ^d^	0.289
Female	14 (50.0)	21 (77.8)		
Male	14 (50.0)	6 (22.2)		
BMI, kg/m^2^	27.59 ± 4.28	28.17 ± 4.50	0.625 ^a^	0.13
Symptom duration, months	5.00 [4.00–6.00]	6.00 [4.00–8.00]	0.064 ^b^	0.25
Affected side, *n* (%)			0.919 ^c^	0.014
Left	10 (35.7)	10 (37.0)		
Right	18 (64.3)	17 (63.0)		
Dominant side, *n* (%)			1.000 ^d^	0.005
Left	2 (7.1)	2 (7.4)		
Right	26 (92.9)	25 (92.6)		
VAS baseline	7.00 [5.25–8.00]	6.00 [5.00–8.00]	0.446 ^b^	0.10
Grip strength baseline, kg	10.50 [6.25–18.00]	9.00 [6.00–12.00]	0.339 ^b^	0.13
Pinch strength baseline, kg	3.75 [3.00–5.00]	3.50 [2.50–4.50]	0.377 ^b^	0.12
QuickDASH baseline	50.61 ± 18.52	52.21 ± 13.17	0.715 ^a^	0.10

Data are presented as mean ± standard deviation, median [interquartile range], or *n* (%), as appropriate. ^a^ Independent samples *t*-test. ^b^ Mann–Whitney U test. ^c^ Chi-square test. ^d^ Fisher’s exact test. Effect sizes are reported as Hedges’ g for normally distributed continuous variables, r for non-normally distributed continuous variables, and Cramer’s V for categorical variables. BMI: body mass index; VAS: Visual Analog Scale; QuickDASH: Quick Disabilities of the Arm, Shoulder and Hand.

**Table 3:** Within- and between-group changes in clinical outcomes.

Outcome	Time	Mesotherapy Group *n* = 28		Saline Group *n* = 27		Between-Group Statistic	*p*-Value	Effect Size
		Mean ± SD	Median [Q1–Q3]	Mean ± SD	Median [Q1–Q3]			
**VAS**	Pre	6.75 ± 1.74	7.00 [5.25–8.00]	6.48 ± 1.63	6.00 [5.00–8.00]			
	Post	2.50 ± 1.69	2.00 [2.00–3.00]	4.00 ± 1.78	4.00 [3.00–5.00]			
	Δ	4.25 ± 2.08	4.00 [2.25–6.00]	2.48 ± 1.67	2.00 [1.00–4.00]	t = 3.463	0.001 ^a^	0.92
	Within-group statistic	t = 10.792		t = 7.709				
	Within-group *p*	<0.001 ^c^		<0.001 ^c^				
	Within-group effect size	dz = 2.04		dz = 1.48				
**Grip strength, kg**	Pre	11.64 ± 6.49	10.50 [6.25–18.00]	10.37 ± 7.43	9.00 [6.00–12.00]			
	Post	15.50 ± 7.17	17.00 [10.00–22.00]	10.78 ± 6.15	8.00 [7.00–12.00]			
	Δ	3.86 ± 3.74	4.00 [2.00–6.00]	0.41 ± 3.99	1.00 [0.00–3.00]	Z = −3.587	<0.001 ^b^	0.48
	Within-group statistic	Z = −3.696		Z = −1.012				
	Within-group *p*	<0.001 ^d^		0.312 ^d^				
	Within-group effect size	r = 0.70		r = 0.19				
**Pinch strength, kg**	Pre	3.86 ± 1.28	3.75 [3.00–5.00]	3.61 ± 1.24	3.50 [2.50–4.50]			
	Post	4.34 ± 1.45	4.50 [3.13–5.38]	4.17 ± 1.23	4.00 [3.50–5.00]			
	Δ	0.48 ± 1.04	0.00 [0.00–1.00]	0.56 ± 1.29	0.50 [0.00–1.50]	Z = −0.737	0.461 ^b^	0.10
	Within-group statistic	Z = −2.193		Z = −2.322				
	Within-group *p*	0.028 ^d^		0.020 ^d^				
	Within-group effect size	r = 0.41		r = 0.45				
**QuickDASH**	Pre	50.61 ± 18.52	55.68 [35.23–63.06]	52.21 ± 13.17	54.54 [42.90–62.60]			
	Post	29.65 ± 16.75	27.80 [12.90–44.25]	32.44 ± 15.57	34.09 [20.45–43.68]			
	Δ	20.96 ± 18.64	16.25 [8.03–27.20]	19.77 ± 16.16	19.12 [4.30–31.82]	Z = −0.135	0.893 ^b^	0.02
	Within-group statistic	Z = −4.623		Z = −4.205				
	Within-group *p*	<0.001 ^d^		<0.001 ^d^				
	Within-group effect size	r = 0.87		r = 0.81				

Data are presented as mean ± standard deviation and median [interquartile range]. Δ indicates change from baseline to post-treatment; positive Δ values indicate clinical improvement for all outcomes. ^a^ Independent samples *t*-test for between-group comparison of change scores. ^b^ Mann–Whitney U test for between-group comparison of change scores. ^c^ Paired samples *t*-test for within-group comparison. ^d^ Wilcoxon signed-rank test for within-group comparison. Effect sizes are reported as Hedges’ g for independent samples *t*-test, Cohen’s dz for paired samples *t*-test, and r for non-parametric tests. VAS: Visual Analog Scale; QuickDASH: Quick Disabilities of the Arm, Shoulder and Hand; SD: standard deviation; Q1–Q3: first and third quartiles.

**Table 4:** Adjusted between-group differences in post-treatment outcomes.

Outcome	Adjusted Mean Difference	95% CI	F	*p*-Value	Partial η^2^
VAS post-treatment	−1.91	−2.83 to −0.98	17.189	<0.001	0.260
Grip strength post-treatment	4.22	1.92 to 6.52	13.610	0.001	0.217
Pinch strength post-treatment	−0.06	−0.73 to 0.61	0.035	0.853	0.001
QuickDASH post-treatment	−2.95	−12.00 to 6.10	0.430	0.515	0.009

CI: confidence interval; VAS: Visual Analog Scale; QuickDASH: Quick Disabilities of the Arm, Shoulder and Hand. Values represent adjusted mean differences between the mesotherapy and saline groups. Adjusted mean differences were calculated as mesotherapy minus saline. Models were adjusted for the corresponding baseline outcome value, age, sex, and symptom duration. Negative adjusted mean differences indicate lower post-treatment scores in the mesotherapy group compared with the saline group. For VAS and QuickDASH, lower scores indicate better outcomes; for grip and pinch strength, higher scores indicate better outcomes. The homogeneity of error variances assumption was not met for the adjusted grip strength model (Levene’s F = 2.98, *p* = 0.090); this result was therefore verified using heteroscedasticity-consistent (HC3) robust standard errors and a nonparametric bootstrap (5000 resamples), both of which confirmed a statistically significant adjusted between-group difference (HC3: 95% CI 1.60 to 6.84, *p* = 0.002; bootstrap: 95% CI 1.60 to 6.47, *p* = 0.002), consistent with the non-parametric change-score analysis (Supplementary Table S1).

## Data Availability

The data supporting the findings of this study are available from the corresponding author upon reasonable request, subject to ethical and privacy restrictions.

## Supplementary Materials

The following supporting information can be downloaded at: https://www.mdpi.com/article/10.3390/jcm15218339/s1, Table S1: Sensitivity analysis of the adjusted grip strength model; Table S2: Responder analysis based on clinically meaningful improvement; Table S3: Spearman correlation analysis of clinical changes and symptom duration.

## Abbreviations

The following abbreviations are used in this manuscript:

VAS	Visual Analog Scale
QuickDASH	Quick Disabilities of the Arm, Shoulder and Hand
BMI	Body Mass Index
LE	Lateral Epicondylitis
CI	Confidence Interval
SD	Standard Deviation
HC3	Heteroscedasticity-Consistent (robust standard errors)
ESWT	Extracorporeal Shock Wave Therapy
MCID	Minimal Clinically Important Difference
PRTEE	Patient-Rated Tennis Elbow Evaluation
ANCOVA	Analysis of Covariance
