# Supplementary material for: Short-Term Clinical Outcomes of Mesotherapy vs. Saline Injection in Patients with Lateral Epicondylitis: A Retrospective Comparative Study
Source: J Clin Med. 2026 Oct 26;15(21):8339. doi: 10.3390/jcm15218339 (PMC13764614; doi:10.3390/jcm15218339)
Supplement: Supplementary file 1 [file jcm-15-08339-s001.zip › jcm-4574510-supplementary.pdf]

Supplementary Materials

Supplementary Table S1. Sensitivity analysis of the adjusted grip strength model

Method	Adjusted mean difference	95% CI	*p*-value
Original (classical) ANCOVA	4.22	1.92 to 6.52	0.001
Heteroscedasticity-consistent (HC3) robust standard errors	4.22	1.60 to 6.84	0.002
Nonparametric bootstrap (5000 resamples)	4.11	1.60 to 6.47	0.002
Quade rank-ANCOVA (fully nonparametric)	—	—	0.005

CI: confidence interval; HC3: heteroscedasticity-consistent robust standard errors; ANCOVA: analysis of covariance.

Supplementary Table S2. Responder analysis based on clinically meaningful improvement

Outcome	Responder definition	Mesotherapy group n=28	Saline group *n*=27	*p*-value	Effect size
VAS	≥1.5-point reduction	27 (96.4%)	19 (70.4%)	0.012[a]	Cramer's V=0.352
QuickDASH	≥15.91-point reduction	14 (50.0%)	17 (63.0%)	0.333[b]	Cramer's V=0.131

Data are presented as n (%). Responder was defined as achieving clinically meaningful improvement from baseline to post-treatment. VAS responder was defined as a ≥1.5-point reduction in pain intensity, and QuickDASH responder was defined as a ≥15.91-point reduction in QuickDASH score, based on previously reported MCID thresholds. [a] Fisher's exact test. [b] Chi-square test.

Supplementary Table S3. Spearman correlation analysis of clinical changes and symptom duration

Variables	Spearman's rho	*p*-value
Symptom duration – VAS change	0.094	0.493
Symptom duration – Grip strength change	-0.163	0.233
Symptom duration – QuickDASH change	-0.135	0.326
VAS change – Grip strength change	0.396	0.003
VAS change – Pinch strength change	0.131	0.339
VAS change – QuickDASH change	0.084	0.541

Spearman's rho was used for correlation analyses. Positive change values indicate clinical improvement for all outcomes. VAS change was calculated as baseline VAS minus post-treatment VAS; grip and pinch strength changes were calculated as post-treatment minus baseline values; QuickDASH change was calculated as baseline QuickDASH minus post-treatment QuickDASH.
